# Translocation of the ABC transporter ABCD4 from the endoplasmic reticulum to lysosomes requires the escort protein LMBD1

**DOI:** 10.1038/srep30183

**Published:** 2016-07-26

**Authors:** Kosuke Kawaguchi, Takumi Okamoto, Masashi Morita, Tsuneo Imanaka

**Affiliations:** 1 Department of Biological Chemistry, Graduate School of Medicine and Pharmaceutical Sciences, University of Toyama, 2630 Sugitani, Toyama 930-0194, Japan

## Abstract

We previously demonstrated that ABCD4 does not localize to peroxisomes but rather, the endoplasmic reticulum (ER), because it lacks the NH_2_-terminal hydrophilic region required for peroxisomal targeting. It was recently reported that mutations in *ABCD4* result in a failure to release vitamin B_12_ from lysosomes. A similar phenotype is caused by mutations in *LMBRD1*, which encodes the lysosomal membrane protein LMBD1. These findings suggested to us that ABCD4 translocated from the ER to lysosomes in association with LMBD1. In this report, it is demonstrated that ABCD4 interacts with LMBD1 and then localizes to lysosomes, and this translocation depends on the lysosomal targeting ability of LMBD1. Furthermore, endogenous ABCD4 was localized to both lysosomes and the ER, and its lysosomal localization was disturbed by knockout of *LMBRD1*. To the best of our knowledge, this is the first report demonstrating that the subcellular localization of the ABC transporter is determined by its association with an adaptor protein.

The superfamily of ATP-binding cassette (ABC) transporters comprises membrane-bound proteins that catalyze the ATP-dependent transmembrane transport of a wide variety of substrates^1^. The human ABC transporter family currently comprises 49 members classified into seven subfamilies, A to G, based on structural organization and amino acid homology^2^,^3^. To date, four ABC proteins have been identified in mammals and classified into “subfamily D”^4^,^5^,^6^,^7^. Each ABCD protein is half-sized and is thus suggested to function as a homodimer^8^. ABCD1‒3 are localized to peroxisomes and suggested to be involved in the transport of very long or long chain fatty acids into these organelles^9^,^10^,^11^.

ABCD4 was identified by a homology search for ABCD1- and ABCD3-related sequences in a database of expressed sequence tags^7^. Human *ABCD4* gene encodes a 606 amino acid protein, the sequence of which has significant amino acid identity homology (25‒27%) with peroxisomal ABC proteins. In an early study, Shani *et al.* demonstrated that ABCD4, which has a molecular mass of 73 kDa, which is larger than that of ABCD3, was localized to rat hepatic peroxisomes using an antibody against the COOH-terminal 194 amino acids of human ABCD4 fused to NH_2_-terminal maltose-binding protein^7^. In addition, we prepared CHO cells stably expressing human ABCD4 in order to investigate the function of ABCD4. We found that the molecular mass of the ABCD4 expressed in CHO cells was 67 kDa, and that ABCD4 is an endoplasmic reticulum (ER) resident-protein, not a peroxisome-resident protein^12^. As ABCD4 lacks the NH_2_-teminal hydrophobic region that is responsible for the targeting to peroxisomes, ABCD4 is co-translationally inserted into the ER membranes through signal recognition particles^12^. Nevertheless, the function of ABCD4 still remained unknown at that time.

Recently, ABCD4 was suggested to be involved in a newly identified inherited disease affecting vitamin B_12_ (cobalamin) metabolism^13^. In humans, cobalamin forms a complex with transcobalamin in the blood stream. This complex specifically binds to the transcobalamin receptor and is taken up into lysosomes by endocytosis. Cobalamin is then released into the cytosol and converted into two active cofactors: methylcobalamin (MeCbl), required by the cytosolic enzyme methionine synthase (MS) that catalyzes the methylation of homocysteine to methionine, and adenosylcobalamin (AdoCbl), required by the mitochondrial enzyme methylmalonyl-CoA mutase (MCM) that converts methylmalonyl-CoA to succinyl-CoA. Nine inherited defects in the intracellular processing of cobalamin are known, designated cblA to cblG, cblJ and mut. These defects result in the accumulation of methylmalonic acid, homocysteine or both, which in turn leads to methylmalonic aciduria and/or isolated homocystinuria^14^. Mutation of ABCD4, which is known as the cblJ complementation group, results in the failure of the release of cobalamin from lysosomes. A similar phenotype in patients within the cblF group is caused by mutations of LMBD1, a lysosomal membrane protein^15^.

LMBD1 is a 62-kDa protein containing 540 amino acids, with nine putative transmembrane domains (TMDs). LMBD1 shares significant homology with the limb region protein^16^ and lipocalin-1 interacting membrane receptor^17^. Although the function of the limb region protein has not yet been determined, the lipocalin-1 interacting membrane receptor is suggested to be involved in internalization of β-lactoglobulin, a member of the lipocalin protein family^18^. Recently, it was demonstrated that a small portion of LMBD1 is present on plasma membranes and functions as a specific adaptor for the clathrin-mediated endocytosis of the insulin receptor^19^.

Mutations of ABCD4 and LMBD1 result in a quite similar phenotype. This suggests these two proteins function as a complex. Indeed, it has been demonstrated that ABCD4 and LMBD1 form complex *in vitro*^20^. In the case of cobalamin transport in *E. coli*, the ABC protein BtuCD facilitates the uptake of cobalamin. We therefore postulated that ABCD4 is a transporter of cobalamin and forms a complex with LMBD1 for the proper targeting or functioning, or both. In this study, we show that LMBD1 associates with ABCD4 on the ER membranes and supports the translocation of ABCD4 to lysosomes.

## Results

### Subcellular localization of ABCD4 with or without the expression of LMBD1

**Figure 1:**
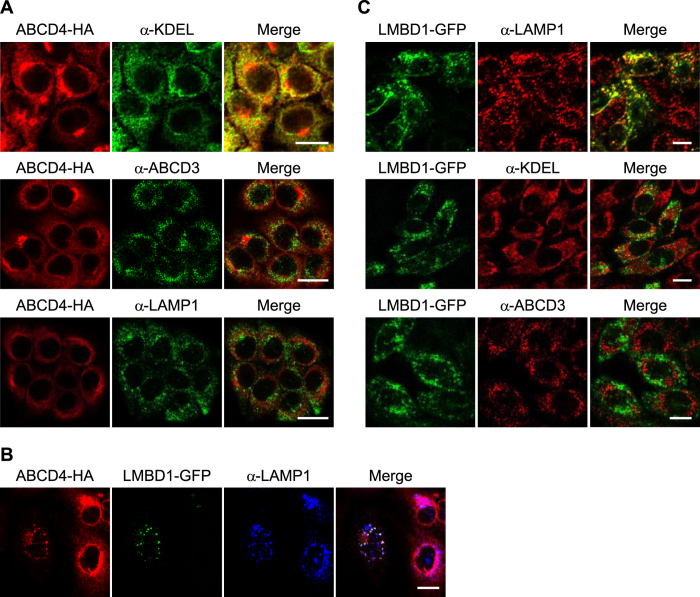
Subcellular localization of the ABCD4-HA and LMBD1-GFP expressed in mammalian cells. (**A**) HuH7 cells stably expressing ABCD4-HA were stained with an anti-HA antibody. The ER, peroxisomes and lysosomes were labeled with an anti-KDEL, anti-ABCD3 and anti-LAMP1 antibody, respectively. Bar, 20 μm. (**B**) GFP-fused LMBD1 was transiently expressed in HuH7 cells stably expressing ABCD4-HA. The subcellular localization of ABCD4-HA and LMBD1-GFP was compared with that of lysosomes labeled with anti-LAMP1. Bar, 20 μm. (**C**) LMBD1-GFP was stably expressed in CHO cells. The distribution of LMBD1-GFP was compared with that of lysosomes, ER and peroxisomes stained with anti-LAMP1, anti-KDEL or anti-ABCD3, respectively. Bar, 10 μm.

We previously reported that ABCD4 in fusion with HA (ABCD4-HA) transiently expressed in CHO cells is localized to the ER^12^. To examine whether the subcellular localization of ABCD4 is shifted by the expression of LMBD1, we prepared human hepatoma HuH7 cells stably expressing ABCD4-HA, and then transiently expressed LMBD1 fused with GFP (LMBD1-GFP) in the cells. We first examined the subcellular localization of ABCD4-HA. As shown in Fig. 1A, ABCD4-HA exhibited a reticulum-like distribution that was superimposable on that of ER-resident proteins possessing the COOH-terminal KDEL, but not LAMP1, a lysosomal marker protein, or ABCD3, a peroxisomal marker protein. These data show that ABCD4-HA is present on the ER in HuH7 cells. Subsequently, we transiently expressed LMBD1-GFP in the cells. As a result, the distribution of ABCD4-HA was drastically changed to a pattern of small dots and ABCD4-HA displayed a similar distribution pattern for LMBD1. Both distribution patterns coincided with that of LAMP1 (Fig. 1B), although some of the ABCD4 positive particles did not display any fluorescence for LMBD1 or LAMP1. This suggests that LMBD1 associates with ABCD4 at the ER membrane and transports ABCD4 to lysosomes.

To confirm the localization of LMBD1 itself, we prepared CHO cells stably expressing LMBD1-GFP. The molecular mass of the expressed LMBD1-GFP was ~150 kDa (Fig. S1), which is bigger than the molecular mass deduced from the amino acid sequence (89 kDa). As LMBD1 possesses six putative glycosylation sites, LMBD1 seems likely to be glycosylated, as seen in COS7 cells expressing LMBD1-EGFP^15^. The distribution of LMBD1-GFP coincided with that of LAMP1, i.e. having neither ER nor peroxisomal marker proteins (Fig. 1C), indicating that LMBD1-GFP is localized in lysosomes.

### Interaction between ABCD4 and LMBD1

**Figure 2:**
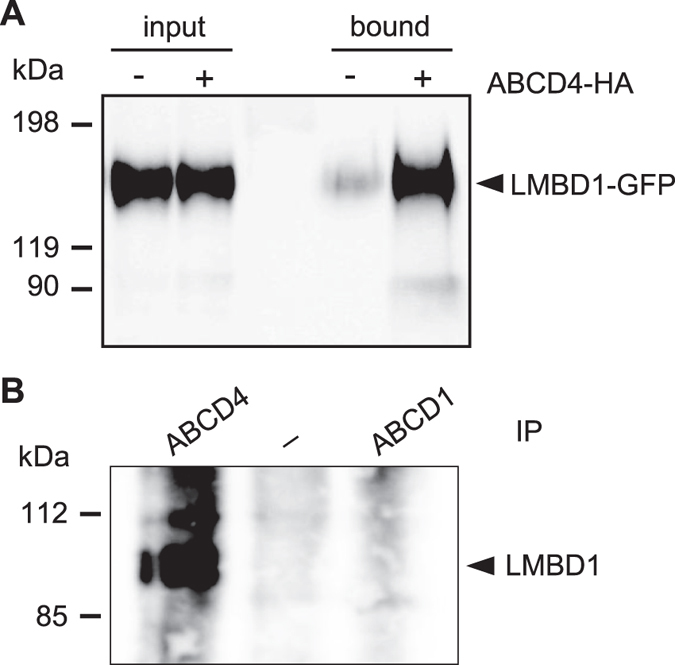
The interaction between ABCD4 and LMBD1. (**A**) Cell homogenates were prepared from LMBD1-GFP expressing CHO cells with or without the transfection of ABCD4-HA. After treatment with 1% digitonin, solubilized proteins were subjected to immunoprecipitation using an anti-HA antibody. Co-precipitated proteins were analyzed by SDS-PAGE followed by immunoblotting using an anti-GFP antibody. (**B**) Interaction with endogenous ABCD4 and LMBD1. Crude membranes from HEK293 cells were solubilized with 0.5% β-DDM. Immunoprecipitation was performed with an anti-ABCD4 or anti-ABCD1 antibody. Elutes were analyzed on SDS-PAGE followed by immunoblotting with an anti-LMBD1 antibody.

**Figure 3:**
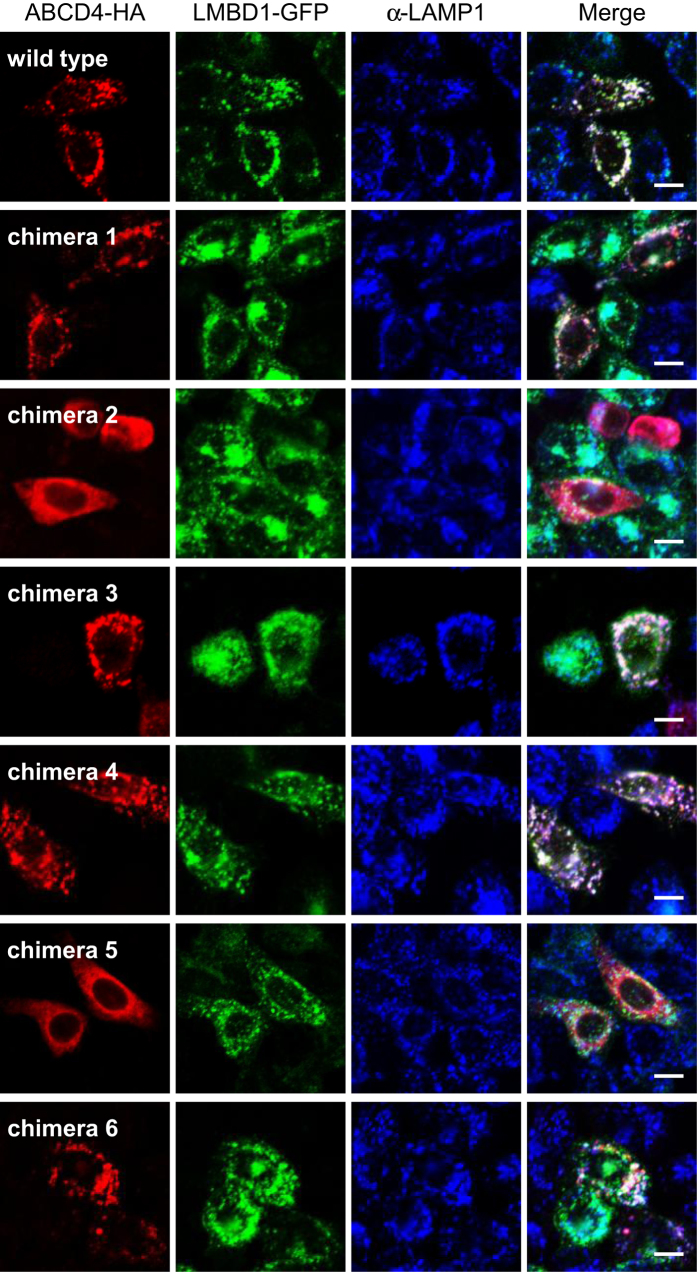
Subcellular localization of the chimeric ABCD4-HA co-expressed with LMBD1-GFP in CHO cells. The distribution of chimeric ABCD4 that was detected by immunofluorescence staining with an anti-HA antibody was compared with the localization of LMBD1-GFP. Bar, 10 μm.

To examine whether ABCD4 interacts with LMBD1 in cells, we performed co-immunoprecipitation of ABCD4-HA with LMBD1-GFP. CHO cells stably expressing LMBD1 were transfected with pcDNA3.1-ABCD4-HA. As a control, the CHO cells were transfected with pcDNA3.1. The post-nuclear supernatant (PNS) fractions were prepared from the CHO cells and solubilized with 1% digitonin. These samples were subjected to co-immunoprecipitation using an anti-HA antibody. As shown in Fig. 2A, LMBD1-GFP was co-immunoprecipitated with ABCD4-HA. In addition, the distribution of ABCD4-HA appeared as small dots and was superimposable on the distribution pattern of LMBD1-GFP in the cells (Fig. 3, wild type). Furthermore, it was confirmed using an anti-GFP that ABCD4-HA was co-immunoprecipitated with LMBD1-GFP (Fig. S2). These results show that ABCD4 is able to form a complex with LMBD1 in cells.

**Figure 4:**
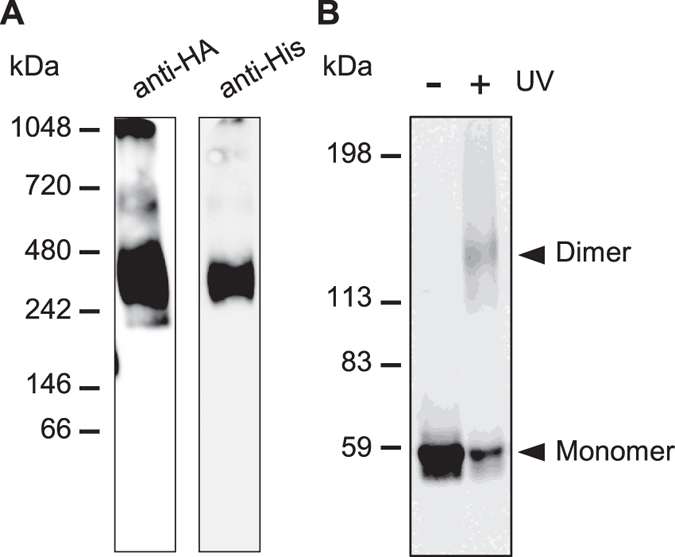
The assembly of ABCD4 on ER membranes. (**A**) PNS was prepared from HuH7 cells expressing both ABCD4-HA and ABCD4-His. After treatment with 0.5% β-DDM, the assembly of ABCD4 was analyzed by BN-PAGE followed by immunoblotting with an anti-HA (left) or anti-His (right) antibody. (**B**) Photocrosslinking of ABCD4-HA was performed with SDA and UV irradiation. ABCD4-HA was detected by immunoblot analysis with an anti-HA antibody.

Next, to clarify whether LMBD1 binds to the ABCD4 monomer or dimer, we transiently expressed ABCD4-His in HuH7 cells stably expressing ABCD4-HA, then performed Blue Native PAGE (BN-PAGE) and co-immunoprecipitation. The PNS fraction was prepared from the HuH7 cells and solubilized with 0.5% β-DDM. The sample was subjected to BN-PAGE followed by immunoblot analysis using an anti-HA or anti-His antibody. As shown in Fig. 4A, ABCD4-His appeared to be comparable in size to ABCD4-HA. However, the molecular weight of these bands was approximately 350 kDa. In addition, there was no band corresponding to the ABCD4 monomer (Fig. 4A) and ABCD4-His was co-immunoprecipitated with ABCD4-HA (Fig. S3). These results suggest that ABCD4-HA and ABCD4-His are part of the same complex and interacted with each other, while ABCD4 itself forms an oligomer. With regard to the ABCD4 structure, Dema *et al.* showed that the detergent-solubilized ABCD4 expressed in Sf9 insect cells existed mainly as a 350 kDa complex, and suggested that the complex was a homodimer by chemical crosslinking and multi-angle light scattering^20^. In fact, ABCD4-HA transiently expressed in human embryonic kidney HEK293 cells displayed a ~140 kDa complex consistent in mass with dimeric ABCD4 after photo crosslinking (Fig. 4B). Therefore, it is deduced that LMBD1 binds to not an ABCD4 monomer, but a dimer.

### The critical region of ABCD4 for translocation

LMBD1 is a hydrophobic protein that has nine TMDs and is embedded in the lysosomal membrane. ABCD4 might interact with LMBD1 through each of their TMD regions. To examine this possibility, we prepared chimeric ABCD4 proteins that were exchanged in terms of the corresponding putative transmembrane helix with ABCD1, based on the secondary structure of the eukaryotic P-glycoprotein homolog CmABCB1 (the crystal structure of which was recently resolved at 2.6-Å^21^). This was done in order to maintain the 3D structure of ABCD4 to the greatest extent possible (Fig. S4). We initially confirmed that endogenous ABCD1 does not interact with LMBD1 by co-immunoprecipitation using an anti-ABCD1 antibody. ABCD4 and ABCD1 were solubilized with 0.5% β-DDM from the membrane fraction of HEK293 cells, and co-immunoprecipitated with an anti-ABCD4 or anti-ABCD1 antibody followed by immunoblot analysis with an anti-LMBD1 antibody. As shown in Fig. 2B, LMBD1 was detected in the fraction that was co-immunoprecipitated with anti-ABCD4, not anti-ABCD1, indicating that ABCD1 does not interact with LMBD1. In contrast, ABCD4 formed a complex with LMBD1.

Subsequently, we constructed ABCD4 chimeras 1‒6 and examined the localization of chimeric ABCD4s in CHO cells stably expressing LMBD1-GFP. The wild type ABCD4 co-expressed with LMBD1 exhibited a punctate distribution that was superimposable on the distribution pattern of LMBD1 (Fig. 3). The distribution patterns of the ABCD4 chimeras 1, 3, 4 and 6 also displayed the same pattern as LMBD1. However, the ABCD4 chimeras 2 and 5 did not exhibit a punctate pattern, but rather, a reticulum-like distribution pattern that was not superimposable on LMBD1 (Fig. 3). These results reveal that the regions around transmembrane helix 2 and 5 of ABCD4 are critically important for the translocation of ABCD4 to lysosomes along with LMBD1.

**Figure 5:**
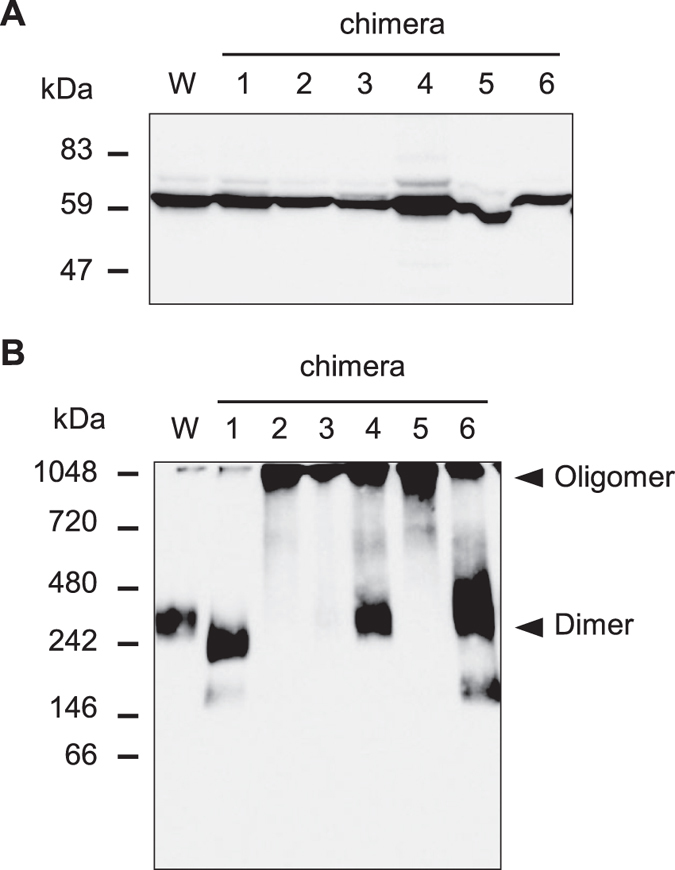
The assembly of chimeric ABCD4 in CHO cells. Cell homogenates were prepared from CHO cells transiently expressing wild type or chimeric ABCD4-HA alone. (**A**) SDS-PAGE analysis followed by immunoblotting with an anti-HA. (**B**) After treatment with 0.5% β-DDM, the assembly of ABCD4 was analyzed by BN-PAGE followed by immunoblotting with an anti-HA antibody.

The interaction of TMDs with half size ABC transporters is important for dimerization. In the case of the half transporter CmABCB1, the TMD2 in one of the CmABCB1 regions is associated with the TMD5 in the other CmABCB1^21^. Therefore, to examine whether the regions around TM2 and 5 are involved in either the interaction with LMBD1 or the homodimer formation of ABCD4, the PNS was prepared from CHO cells transiently expressing wild type or chimeric ABCD4-HA and subjected to BN-PAGE. Prior to the experiment, it was confirmed that the wild type and each chimeric ABCD4 exhibited equal band mobility on SDS-PAGE (Fig. 5A). After BN-PAGE, the wild type ABCD4 exhibited a complex of ~350 kDa and the respective size of chimeras 1, 4 and 6 was comparable with that of the wild type ABCD4 (Fig. 5B). On the other hand, chimeras 2 and 5 existed as a bigger complex, respectively, indicating higher-order oligomer formation. This suggests that incorrect dimer formation of ABCD4 disturbs LMBD1 lysosome targeting. However, chimera 3 was translocated from the ER to lysosomes in CHO cells stably expressing LMBD1 (Fig. 3), even though it also exhibited higher-order oligomer formation (Fig. 5B). Therefore, the regions around TM2, 5 and 3 are suggested to be critical for the correct dimerization of ABCD4, while a putative region of ABCD4 that interacts with LMBD1 might be masked by the exchange of the regions around TM2 and 5.

### The mistargeting of mutant LMBD1 affects the distribution of ABCD4

**Figure 6:**
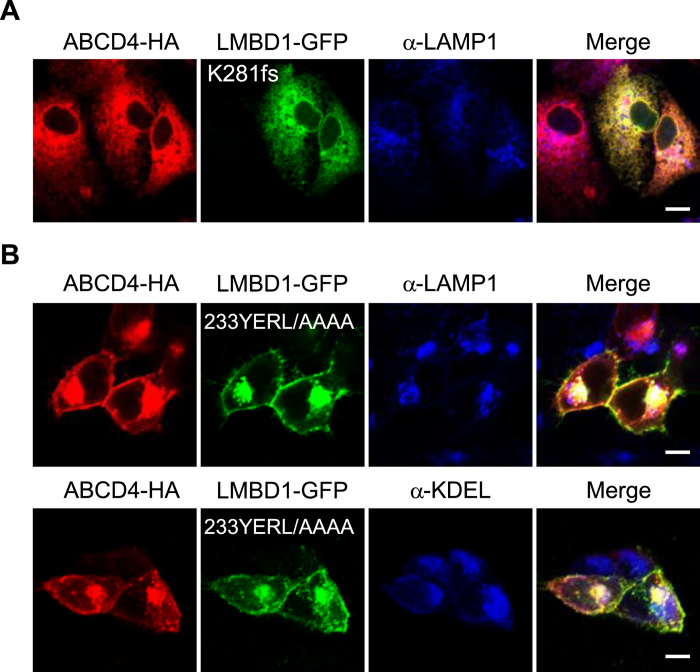
Subcellular localization of ABCD4-HA co-expressed with mutant LMBD1-GFP. (**A**) The distribution of ABCD4-HA and LMBD1(K281fs)-GFP was compared with that of lysosomes stained with anti-LAMP1 in HuH7 cells. (**B**) HEK293 cells transiently coexpressing ABCD4-HA and LMBD1(233YERL/AAAA)-GFP were stained with an anti-HA antibody. The ER and lysosomes were labeled with anti-KDEL and anti-LAMP1, respectively. Bar, 10 μm.

To determine whether the translocation of ABCD4 from the ER to lysosomes depends on the lysosomal targeting ability of LMBD1, we expressed mutant LMBD1 in HuH7 cells stably expressing ABCD4-HA. Among six mutants found with cobalamin deficiency^22^, we first attempted to express LMBD1(D469fs)-GFP, which possesses the mutation closest to the COOH-terminal, examining the subcellular localization pattern by immunofluorescence. However, no fluorescence of LMBD1(D469fs)-GFP was detected, presumably because of the rapid degradation of the mutant LMBD1. Next, we chose LMBD1(K281fs)-GFP, which has its COOH-terminus exposed to the cytosol, because the COOH-terminus of LMBD1(D469fs)-GFP is located inside of the lysosome. In the case of LMBD1(K281fs)-GFP, it was successfully expressed in HuH7 cells and the distribution of the mutant LMBD1 was overlapped with the ER and not the lysosome membrane (Fig. S5). When LMBD1(K281fs)-GFP was expressed in HuH7 cells stably expressing ABCD4-HA, the distribution of ABCD4 did not coincide with that of lysosomes and overlapped with that of LMBD1(K281fs) on the ER membrane (Fig. 6A). Concerning the targeting of LMBD1 to lysosomes, the signal has not yet been elucidated at present. However, recently a small region of LMBD1 was shown to be located on plasma membranes^19^. As LMBD1 possesses two putative AP-2 binding motifs, mutation of these motifs might affect the subcellular localization of LMBD1. Therefore, we prepared mutant LMBD1 with two AP-2 binding motifs (233YERL/AAAA and 295WTKF/AAAA) and examined the intracellular localization. We transiently expressed both ABCD4-HA and LMBD1(233YERL/AAAA) or LMBD1(295WTKF/AAAA), respectively, in HEK293 cells. When LMBD1(295WTKF/AAAA) and ABCD4-HA were co-expressed, the distribution of LMBD1(295WTKF/AAAA) coincided with that of LAMP1 as well as that of the wild type LMBD1, and ABCD4-HA was also localized on lysosomes (Fig. S6). On the other hand, a part of LMBD1(233YERL/AAAA) appeared to exist as aggregates in the cells, but the other LMBD1(233YERL/AAAA) appeared to be localized to cell surface (the plasma membrane) (Fig. 6B). This shows that LMBD1(233YERL/AAAA) was able to exit from the ER, but was unable to localize on lysosomes. Furthermore, the distribution pattern of ABCD4-HA was superimposable on LMBD1(233YERL/AAAA) in the cells (Fig. 6B). These results demonstrate that the translocation of ABCD4 from the ER to lysosomes depends on the translocation of LMBD1.

### The subcellular localization of endogenous ABCD4 in HEK293cells with or without endogenous LMBD1

As the expression level of endogenous ABCD4 in mammalian cells is quite low, it is impossible at present to characterize the subcellular localization of ABCD4 by immunofluorescence microscopy. Thus, we prepared the PNS fraction from HEK293 and immunoblot analysis was performed after concentrating the membrane proteins by Na_2_CO_3_ treatment. The band corresponding to endogenous ABCD4 was determined by comparing it with the mobility of the overexpressed band of ABCD4 that did not bear a tag in CHO cells^12^ (Fig. S7C). We then prepared *LMBRD1* knockout HEK293 cells using the CRISPR/Cas9 system^23^. Mutation of the *LMBRD1* gene was confirmed by sequencing, and the absence of LMBD1 was validated by immunoblot analysis (Fig. S7A,B). In the *LMBRD1* knockout cells, almost the same amount of ABCD4 was present (Fig. S7C).

**Figure 7:**
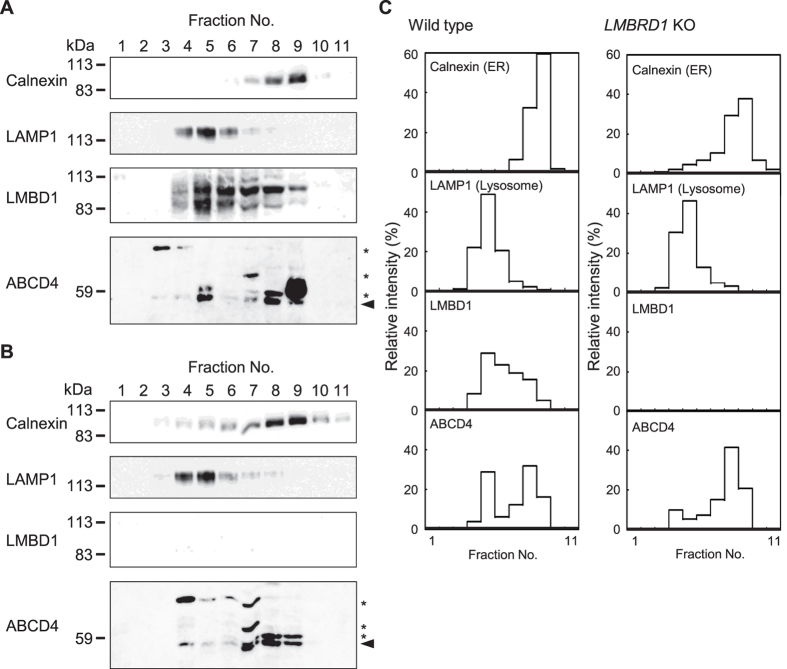
Subcellular localization of endogenous ABCD4. Gradient separation of PNS of the wild type (**A**) or *LMBRD1* knockout (**B**) HEK293 cell homogenate was followed by immunoblotting with antibodies against calnexin (an ER marker), LAMP1 (a lysosomal marker), ABCD4 or LMBD1. The asterisk indicates a non-specific signal. (**C**) The distribution of each enzyme was plotted by quantitating the signal intensities using the image analysis software Image J.

Subsequently, we investigated the subcellular distribution of endogenous ABCD4 in HEK293 cells by equilibrium iodixanol density gradient centrifugation. Isolated subcellular fractions were subjected to immunoblot analysis and bands corresponding to calnexin, LAMP1, LMBD1 and ABCD4 were quantitated by the image analysis software Image J^24^. As shown in Fig. 7A,B, the distribution of the lysosomal marker LAMP1 was well separated from that of the ER marker calnexin in both the wild type and *LMBRD1* knockout HEK293 cells. LMBD1 in wild type cells was recovered mainly in fractions 4‒6 as several bands since LMBD1 is glycosylated^15^, while a portion of LMBD1 was distributed in slightly more high-density fractions compared with calnexin. LMBD1 was not detected in *LMBRD1* knockout cells. Under these conditions, the distribution of ABCD4 exhibited two peaks, which coincided with LAMP1 or calnexin in wild type cells. On the other hand, in the *LMBRD1* knockout cells, the ABCD4 which overlapped with LAMP1 was reduced to ~40% and the ABCD4 in fractions 7‒9 was increased (Fig. 7C). These results show that endogenous ABCD4 was localized on both lysosomes and the ER, and that LMBD1 is responsible for the localization of endogenous ABCD4. Taken together, our results support the view that the translocation of ABCD4 from the ER to lysosomes requires, at least in part, the lysosomal membrane protein LMBD1.

## Discussion

Recently, ABCD4 and LMBD1, encoded by *ABCD4* and *LMBRD1* respectively, were shown to be involved in the export of cobalamin from lysosomes into the cytosol^13^,^15^. Mutation of either gene causes vitamin B_12_ deficiency and results in a similar defect of intracellular transport of cobalamin from lysosomes, suggesting ABCD4 and LMBD1 function in a coordinated manner. Deme *et al.* recently purified LMBD1 and ABCD4 from insect Sf9 cells expressing either ABCD4 or LMBD1 and showed the proteins form a complex *in vitro*^20^. Here we investigated the possibility that LMBD1 is involved in the translocation of ABCD4 to lysosomes from the ER.

ABCD4 was originally reported as a peroxisomal ABC transporter^7^. However, we showed that ABCD4 was not a peroxisomal protein, but rather, an ER protein^12^. It was speculated that ABCD4 is present in lysosomes, at least in part, because a defect impairs the transport of cobalamin from lysosomes^13^. The following evidence shows that LMBD1 is important for the targeting of ABCD4 to lysosomes from the ER. First, the ABCD4 stably expressed in HuH7 cells was located in the ER, and the location was dramatically changed from the ER to lysosomes by the co-expression of LMBD1 (Fig. 1). Second, ABCD4 was co-immunoprecipitated with LMBD1 in the CHO cells stably expressing LMBD1 when ABCD4 was transiently expressed in the cells. In addition, endogenous ABCD4 was also co-immunoprecipitated with LMBD1 in HEK293 cells (Fig. 2). Third, the ABCD4 transiently expressed in the CHO cells stably expressing LMBD1 was also localized in lysosomes (Fig. 3). Fourth, mutant LMBD1, which did not localize to lysosomes, was not able to translocate ABCD4 from the ER to lysosomes (Fig. 6). Fifth, endogenous ABCD4 was localized to both the lysosomes and ER, and its lysosomal localization was disturbed by the absence of LMBD1 (Fig. 7).

Regarding the translocation of ABCD4 from the ER to lysosomes associated with LMBD1, we initially thought the regions surrounding TM2 and 5 of ABCD4 to be the most important for the association with LMBD1 based on the evidence that the targeting of ABCD4 chimeras 2 and 5 to lysosomes was impaired (Fig. 3). However, the evidence that the ABCD4 chimeras 2 and 5 are present as a higher-order oligomeric complex suggests that these regions are required for the correct homodimer formation of ABCD4 (Fig. 5B). Incorrect oligomerization might mask a region that is needed for the interaction with LMBD1. Interestingly, ABCD4 chimera 3 was translocated to lysosomes with LMBD1, although it also formed higher weight oligomer. Homodimerization of ABCD4 through the interaction between TMD2 and TMD5 might result in a retention of the region required for the interaction with LMBD1 at the surface of ABCD4. However, the precise structure of ABCD4 has not been determined. Further studies are required to resolve the mechanism underlying this interaction between ABCD4 and LMBD1.

The ABCD4 overexpressed in mammalian cells was localized to the ER (Fig. 1A), as described previously^12^. On the other hand, when ABCD4-RFP was transiently expressed in human immortalized fibroblasts, ABCD4 was localized to lysosomes^13^. These results might be explained by the difference in expression level of LMBD1, which influences the interaction with ABCD4. In the case of HEK293 cells, ABCD4 was distributed to the ER as well as lysosomes (Fig. 7C). Interestingly, LMBD1 in HEK293 cells localized mainly to the lysosomes where LAMP1 is present, but another portion of LMBD1 appeared to localize to LAMP1 negative endomembranes other than the ER (Fig. 7C). A small amount of ABCD4 might exist in such endomembranes. Further studies are required to clarify whether these endomembranes are in endosomal compartment such as late endosomes. In our previous study, we examined the subcellular localization of endogenous ABCD4 in the rat liver using differential centrifugation and concluded that ABCD4 was resident on the ER membrane^12^. However, we are unable to rule out the possibility that disrupted lysosomal membranes were recovered in the fraction containing ABCD4.

In terms of its role in cellular function, LMBD1 is reported to be a specific adaptor for the clathrin-mediated endocytosis of the insulin receptor^19^. Furthermore, the nuclear export signal-interacting protein (NESI), which lacks 73 NH_2_-terminal amino acids of LMBD1, is suggested to facilitate the chromosome region maintenance 1-independent nuclear export of the large form of the hepatitis delta antigen by forming complexes with lamin A/C^25^. Thus, LMBD1 appears to have multiple functions. It is a novel function that LMBD1 has the ability to translocate ABCD4 from the ER to lysosomes. However, a portion of ABCD4 is still retained in the ER membrane in HEK293 cells (Fig. 7B). This might be explained as follows. A portion of the newly synthesized LMBD1 associates with ABCD4 on the ER. This portion interacts with various proteins without any additional association and is transported to lysosomes. Therefore, the efficiency of the interaction between ABCD4 and LMBD1 is determined by the distribution pattern of ABCD4 in the ER and lysosomes.

Two ABC transporters, TAPL (transporter associated with antigen processing-like) and ABCB6, have been found on the lysosomal membrane^26^,^27^,^28^. TAPL constitutes the lysosomal transport machinery which transports cytosolic peptides into lysosomes^26^. TAPL contains an extra NH_2_-terminal transmembrane domain (TMD_0_) which is not required for the transport function, but is essential for the targeting of TAPL to the lysosomal membrane^29^. ABCB6 also possesses NH_2_-terminal TMD_0_. TMD_0_ comprises an independent folding unit, dispensable for catalysis, that also plays a crucial role in the lysosomal targeting of ABCB6^30^. In contrast, ABCD4 comprises the core that is formed by the TMDs and nucleotide binding domains (NBDs) without any accessory domains. This study shows that the translocation of ABCD4 from the ER to lysosomes depends on the lysosomal targeting ability of LMBD1 (Fig. 6). To the best of our knowledge, this is the first study to report that an ABC transporter is transported to a final, destined organelle with the help of an adapter protein.

What is the function of LMBD1 in the transport of cobalamin? The bacterial ABC transporter BtuCD is involved in the import of cobalamin into the cytoplasm across the inner membranes and possesses ATPase activity^31^. ABCC1, a multi-drug resistance-associated protein on the plasma membranes of mammalian cells, is suggested to be involved in the export of cobalamin out of cells^32^. ABCC1 itself also possesses ATPase activity. Based on these findings, we postulate that ABCD4 is composed of transporter units and LMBD1 is an accessory protein. Concerning the transport activity of the mammalian ABC transporters, almost all function as exporters that transport substrate from the cytosol to the exterior of the cell or the lumen of subcellular organelles, which is considered to be an “extracellular space” in eukaryotic cells. At present, ABCA4 is reportedly the only example of a mammalian ABC importer that actively flips *N*-retinylidene-phosphatidylethanolamine from the lumen to the cytoplasmic leaflet of the disc membrane in retinal photoreceptor cells^33^. If ABCD4 actually transports cobalamin as we postulate, ABCD4 would be the first mammalian ABC importer which transports a soluble compound. Consequently, our next study will investigate the transport of cobalamin mediated by ABCD4.

In summary, based on results reported here, we propose a hypothetical model for the targeting of ABCD4 to lysosomes. The newly synthesized ABCD4 is inserted into the ER membrane and assembles into a homodimer. ABCD4 then associates with the newly synthesized lysosomal membrane protein LMBD1 and ABCD4 is transported to the lysosomes. To the best of our knowledge, this is the first reported case in which the subcellular localization of an ABC transporter is determined by an adaptor protein.

## Materials and Methods

### Cell culture conditions and transfection procedure

Human hepatoma HuH7 cells (Riken BRC, Ibaraki, Japan), human embryonic kidney 293 (HEK293) cells (ATCC, Manassas, VA) and Chinese hamster ovary (CHO) cells (Riken BRC) were cultured in Dulbecco’s modified Eagle medium (DMEM) supplemented with 10% fetal bovine serum, 100 units/ml penicillin and 140 μg/ml streptomycin sulfate at 37 °C and 5% CO_2_. All of the transfection procedures were performed using the Effectene Transfection Reagent (Qiagen, Hilden, Germany) according to the manufacturer’s instructions.

### Cloning of *LMBRD1*

To clone the *LMBRD1* gene, total RNA was extracted from HuH7 cells using a NucleoSpin RNA II kit (Qiagen). cDNA was synthesized from 1 μg of total RNA using a ReverTra Ace qPCR RT Kit (Toyobo, Osaka, Japan). A 1.6-kb section of the *LMBRD1* coding region was PCR amplified with the primers Fw-LMBD1-Kpn and Rv-LMBD1-Xho (Table S1) using the cDNA as the template. The resulting product was ligated into the *Eco*RV site of a KS^+^ cloning vector to yield KS-LMBD1. The sequence of *LMBRD1* was confirmed using the Big Dye V.3.1 Terminator Kit (Applied Biosystems, Foster City, CA) in an ABI Prism 3130 sequencer (Applied Biosystems).

### Plasmid construction

All of the primers used in this study are listed in Table S1. The plasmid pBluescript II SK(+)/ABCD4, which contains the human ABCD4 cDNA sequence, was kindly provided by Dr. Gerardo Jimenez-Sanchez (Johns Hopkins University). From this cDNA, the full-length ABCD4 was excised with *Eco*RI and recloned into a pcDNA3.1+ vector at the corresponding site to obtain pcDNA3.1-ABCD4. Next, an HA-tagged ABCD4 expression vector was constructed as follows. A 7.3-kb fragment was amplified by inverse PCR using the primer set Fw-inv-ABCD4-HA/Rv-inv-ABCD4-HA and pcDNA3.1-ABCD4 as the template, and then this fragment was self-ligated using T4 Polynucleotide Kinase (Toyobo) and Ligation high (Toyobo) to form pcDNA3.1-ABCD4-HA.

An expression vector for chimeric ABCD4, which swaps the putative transmembrane (TM) helix 1 between ABCD4 and ABCD1, was constructed as follows. The 7.2-kb fragment lacking the putative TM helix 1 of ABCD4 was amplified by inverse PCR using the primers Fw-D4-invTM1 and Rv-D4-invTM1, with pcDNA3.1-ABCD4-HA as the template. The 0.15-kb fragment coding the putative TM helix 1 of ABCD1 was amplified using the primer set Fw-D1-TM1/Rv-D1-TM1 and pcDNA4/ABCD1^34^ as the template. These two fragments were fused using an In-Fusion PCR cloning kit (TaKaRa, Shiga, Japan) to yield pcDNA3.1-ABCD4-chimera1. All of the chimeric ABCD4 expressing vectors were constructed following the same procedure as described above.

The LMBD1 coding region was excised with *Kpn*I/*Xho*I from KS-LMBD1 and ligated with the 5.4-kb *Kpn*I-*Xho*I fragment of pcDNA3.1 to form pcDNA3.1-LMBD1. The GFP-tagged LMBD1 (LMBD1-GFP) expression vector was constructed as follows. A 7.0-kb fragment was amplified by inverse PCR using the primer set Fw-inv-LMBD-Cla/Rv-inv-LMBD-Cla and pcDNA3.1-LMBD1 as the template. The GFP-coding region was PCR amplified with the primer set Fw-GFP-Cla/Rv-GFP-Cla. These two fragments were digested with *Cla*I and ligated to form pcDNA3.1-LMBD1-GFP. The mutant LMBD1-GFP expression vector was constructed as follows. A 7.7-kb fragment was amplified by inverse PCR using the primer set Fw-LMBD1-del848-851/Rv-LMBD1-del848-851 and pcDNA3.1-LMBD1-GFP as the template, and then this fragment was self-ligated as described above to yield pcDNA3.1-LMBD1-GFP-del848-851. Furthermore, the nonsense region of this plasmid was removed by inverse PCR and self-ligation was performed with the primer set Fw-mutLMBD1-GFP and Rv-LMBD1-K281fs to form pcDNA3.1-LMBD1-GFP-K281fs. An LMBD1-GFP-D469fs expression vector was constructed using the same procedure described above. The AP-2 binding motif mutants of an LMBD1-GFP, 233YERL/AAAA or 295WTKF/AAAA expressing vector were obtained by inverse PCR and self-ligation was performed using the primer sets Fw-LMBD1-233AAAA/Rv-LMBD1-233AAAA and Fw-LMBD1-295AAAA/Rv-LMBD1-295AAAA, respectively, as described above.

### Indirect immunofluorescence

Immunostaining was performed by essentially the same procedure as described previously^12^. Fixed cells were permeabilized in PBS containing 1% (w/v) Triton X-100 for 5 min or 30 μM digitonin for 15 min, washed three times with PBS and incubated with the primary antibodies for 45 min at room temperature. The primary antibodies in this study were used as a combination of a rabbit antibody against HA and a mouse antibody against KDEL, or a mouse antibody against HA and a rabbit antibody against catalase, ABCD3 or LAMP1. Cy3-conjugated goat anti-mouse IgG, Cy3-conjugated goat anti-rabbit IgG, Alexa Fluor 488 goat anti-mouse IgG, Alexa Fluor 488 goat anti-rabbit IgG and Cy5-conjugated goat anti-rabbit IgG were used to mark the primary antibodies. Mitochondria were labeled by the mitochondria-specific dye Mitotracker Red CMXRos (Molecular Probes, Eugene, OR) before fixation. Mitotracker was applied to culture at 250 nM and the cells were incubated for 30 min at 37 °C, and then washed three times with PBS prior to visualization. The cells were mounted in Slowfade^®^ Gold Antifade Reagent (Thermo Fisher Scientific, Waltham, MA) and the samples were observed under a Zeiss LSM780 laser scanning confocal microscope equipped with a Plan-APOCHROMAT 20x, NA 0.8 objective on an inverted microscope Axio Observer Z1(Carl Zeiss, Oberkochen, Germany). Alexa Fluor 488 was excited with a multiline (458, 488, and 514 nm) argon laser. Cy3 and Cy5 were excited with 543 nm and 633 nm HeNe lasers, respectively.

### Co-immunoprecipitation

To examine the interaction between the overexpressed LMBD1 and ABCD4, the post-nuclear supernatant (PNS) fractions were prepared from LMBD1-GFP expressing CHO cells transfected with pcDNA3.1-ABCD4-HA or pcDNA3.1. All of the following procedures were carried out at 4 °C. The PNS was incubated with 1% digitonin for 1 h, then centrifuged at 20,000 × *g* for 20 min. The resulting supernatants were incubated with Anti-HA tag mAb-Magnetic Beads (MBL, Nagoya, Japan) for 1 h. The Anti-HA tag mAb-Magnetic Beads were collected by centrifugation and washed three times with PBS. The immunoprecipitated proteins were subjected to immunoblot analysis.

To examine the interaction between endogenous LMBD1 and ABCD4 or ABCD1, PNS was prepared from HEK293 cells using PBS containing protein inhibitors (PIs) (1 μg/ml Pepstatin A, 1 μg/ml Leupeptin, 1 μg/ml Antipain and 1 μg/ml Chymostatin). All of the following procedures were carried out at 4 °C. PNS was centrifuged at 100,000 × *g* for 30 min to obtain a membrane pellet. The pellet was resuspended with PBS containing PIs and incubated with 0.5% β-DDM for 2 h, then centrifuged at 100,000 × *g* for 30 min. The resulting supernatant was incubated overnight with Protein G-Agarose beads (Sigma Aldrich, St. Louis, MO) pre-incubated with an anti-ABCD4 antibody^12^, anti-ABCD1 antibody^34^ or IgG for 1 h in PBS. The Protein G-Agarose beads were collected by centrifugation and washed three times with PBS. The immunoprecipitated proteins were subjected to immunoblot analysis.

### Blue Native PAGE (BN-PAGE)

To examine whether each ABCD4 forms a dimer or not, PNS was prepared from HuH7 or CHO cells transiently expressing wild type or chimeric ABCD4. Each PNS was incubated with 0.5% β-DDM for 1 h, then mixed with 5 × sample buffer (25 mM Imidazole pH 7.0, 2.5% Coomassie brilliant blue (CBB) G-250, 50% Glycerol). BN-PAGE was performed using 4‒16% polyacrylamide gradient gels. Samples and protein standards (NativeMark^TM^; Life Technologies, Carlsbad, CA) were run in cold anode buffer (25 mM Imidazole pH 7.0) and deeply blue cathode buffer (50 mM Tricine pH7.0, 7.5 mM Imidazole pH7.0, 0.02% CBB G-250) for 45 min at 100 V and then for another 40 min at 150 V. Thereafter, the cathode buffer was replaced with slightly blue cathode buffer (50 mM Tricine pH7.0, 7.5 mM Imidazole pH7.0, 0.002% CBB G-250) and electrophoresis continued for 90 min at 5 mA. The gels were incubated in denaturing buffer (20 mM Tris, 150 mM Glycine, 0.1% SDS) for 10 min and then transferred to PVDF membranes. The membranes were subjected to immunoblot analysis.

### Photocrosslinking

PNS was prepared from HEK293 cells transiently expressing ABCD4-HA using PBS and then incubated with succinimidyl 4,4′-azipentanoate (SDA) (Thermo Fisher Scientific) (final conc. 2 mM) on ice for 2 h. The reaction was stopped by adding Tris-HCl (pH 8.8) at a final concentration of 100 mM and incubated on ice for 15 min. Subsequently, SDA-labeled proteins were photoactivated by UV irradiation at 365 nm for 15 sec.

### CRISPR/Cas9 mutagenesis

Nuclease plasmids for the disruption of the *LMBRD1* gene were constructed as follows. Design of the guide RNAs was carried out using the CRISPR Design Tool (http://crispr.mit.edu) to minimize potential off-target effects. The oligo pairs encoding the 20-nt guide sequences (Table S1) were annealed and ligated into the *Bbs*I site of pSpCas9(BB)-2A-Puro (PX459), which was a gift from Dr. Feng Zhang (Addgene plasmid # 48139)^35^, to yield PX495-lmbrd1. After the transfection of PX495-lmbrd1 into HEK293 cells, clonal cell lines were isolated by dilution. The mutation of *LMBRD1* was confirmed using the Big Dye V.3.1 Terminator Kit in an ABI Prism 3130 sequencer.

### Subcellular fractionation

The PNS was prepared from HEK293 cells using buffer A (0.25 M sucrose, 10 mM Hepes pH7.4, 1 mM EDTA and PIs). Thirty % iodixanol (OptiPrep^TM^; Axis-Shield Density Gradient Media) was diluted using buffer A to prepare 18%, 15% and 12% iodixanol. The same volume of each solution was layered. The tube was carefully rotated to a horizontal position and left for 2 h at 4 °C to form a continuous gradient and then PNS (3 mg) was layered. Subsequently, the samples were spun at 178,000 × *g* for 3 h at 4 °C in a NVT65 near-vertical rotor (Beckman Coulter, Fullerton, CA). The gradients were divided into 11 fractions from the bottom. The fractions were subjected to immunoblot analysis. For the detection of endogenous ABCD4, a half volume of each fraction was concentrated by Na_2_CO_3_ treatment and subsequent ultracentrifugation.

### Immunoblot analysis

After electrotransfer of the proteins to the membranes, the blot was blocked for more than 1 h in 5% skim milk in TBS-T buffer (20 mM Tris-HCl, 137 mM NaCl, and 0.1% Tween-20). The blots were incubated with an anti-ABCD4 antibody (1:5000 dilution), anti-calnexin antibody (Enzo Life Sciences, New York, NY) (1:5000 dilution), anti-LAMP1 antibody (Affymetrix, Santa Clara, CA) (1:5000 dilution) or anti-LMBD1 antibody (Atlas Antibodies, Stockholm, Sweden) (1:3000 dilution) in TBS-T buffer for 1 h with gentle shaking, washed three times in TBS-T, and incubated with an anti-rabbit or anti-mouse IgG HRP-conjugated antibody (1:10000 dilution) in TBS-T buffer for 1 h. The blots were washed three times in TBS-T, and immunoreactive bands were detected using Western Lighting Chemiluminescence Reagent Ultra (Perkin Elmer, Branchburg, NJ).

## Additional Information

**How to cite this article**: Kawaguchi, K. *et al.* Translocation of the ABC transporter ABCD4 from the endoplasmic reticulum to lysosomes requires the escort protein LMBD1. *Sci. Rep.* **6**, 30183; doi: 10.1038/srep30183 (2016).

## Supplementary Material

Supplementary Information
