# Supplementary material for: Translocation of the ABC transporter ABCD4 from the endoplasmic reticulum to lysosomes requires the escort protein LMBD1
Source: Sci Rep. 2016 Jul 26;6:30183. doi: 10.1038/srep30183 (PMC4960490; doi:10.1038/srep30183)
Supplement: Supplementary Information [file srep30183-s1.pdf]

Article

Title:

Translocation of the ABC transporter ABCD4 from the endoplasmic reticulum to lysosomes requires the escort protein LMBD1

Author names and affiliations:

Kosuke Kawaguchi[1], Takumi Okamoto[1], Masashi Morita and Tsuneo Imanaka[*]

Department of Biological Chemistry, Graduate School of Medicine and Pharmaceutical Sciences, University of Toyama, 2630 Sugitani, Toyama 930-0194, Japan

[*]Correspondence and requests for materials should be addressed to T.I.

E-mail: imanaka@pha.u-toyama.ac.jp

Phone: +81-76-434-7545

Fax: +81-76-434-7545;

[1]These authors contributed equally to this work.

Table S1 List of oligonucleotide primers

Primer name	5’- sequence-3’
Fw-inv-ABCD4-HA	CAGACTACGCTGGTTACCCGTACGATGTACCAGACTACGCTTGACTC GAGTCTAGAGGGCCCGTTTAAAC
Rv-inv-ABCD4-HA	GTACATCGTACGGGTAACCAGCGTAGTCTGGTACATCGTACGGGTAT TCCACTTTGAT TCTCATCAGCTC
Fw-D1-TM1	CGGTTCCTGTGGCTCCTGCGGCTGCTGTTC
Rv-D1-TM1	GTCTTTGTTGACGATGCAGCGGGCCAGCC
Fw-D1-TM2	AACAAAGACCCGCGGGCCTTTGGCTGGCAG
Rv-D1-TM2	GTAGAGGCGGTAGGCGTGGGCCACCAGACG
Fw-D1-TM3	CCGGACCAGTCTCTGACGGAGGACGTGG
Rv-D1-TM3	GCACTGGTAGGAAGTCACAGCCACGTCCAG
Fw-D1-TM4	GACCGTGGTGCTGCGGGCCTTCTCGCC
Rv-D1-TM4	GTAGAAAGCGATCTCCTCCGAGTTGGCCAC
Fw-D1-TM5	CATGTGGAGCTGGCCCTGCTACAGCGCTCC
Rv-D1-TM5	GGGGATTGCCACCATGAGCAGGCCCGAG
Fw-D1-TM6	CCCGCAGAGCGCACAGAAGCCTTCACTATTGCCC
Rv-D1-TM6	GTAGCCAGCCAGCTCCGTCACCTCCTTGTACGAC
Fw-D4-invTM1	CTGCATCGTCAACAAAGACTTGGAAGGGTTTAAGACTC
Rv-D4-invTM1	CAGGAGCCACAGGAACCGCTGGAGAAATTG
Fw-D4-invTM2	CACGCCTACCGCCTCTACTTCCGGGGCC
Rv-D4-invTM2	GGCCCGCGGGTCTTTGTTTCCCAGGACC
Fw-D4-invTM3	GTGACTTCCTACCAGTGCTTCCAAAGCACAGGC
Rv-D4-invTM3	CGTCAGAGACTGGTCCGGGTTATCGATGTC
Fw-D4-invTM4	GAGGAGATCGCTTTCTACAGAGCTGGGCATGTGG
Rv-D4-invTM4	GGCCCGCAGCACCACGGTCCCCAGGATG
Fw-D4-invTM5	CTCATGGTGGCAATCCCCATTTTCAGCGGG
Rv-D4-invTM5	GGGCCAGCTCCACATGCCCAGCTCTGTAG
Fw-D4-invTM6	CGGAGCTGGCTGGCTACACGCACAGAATTG
Rv-D4-invTM6	CTTCTGTGCGCTCTGCGGGACTCAGGTCTC
Fw-LMBD1-Kpn	CTGGTACCATGGCGACTTCTGGCG
Rv-LMBD1-Xho	GCATCTCGAGTCAAGCAGAATAGACAGAGG
Fw-LMBD- Cla	GATGACCTCGAGTCTAGAGGGCC
Rv-LMBD- Cla	GATAGCAGAATAGACAGAGGGCTCATCATC
Fw-LMBD1-del1405G	ATGCTCCTGAAGATCAGTGTACTGTTACC
Rv-LMBD1-del1405G	TGCATCACATCTCTTTGGCACAGAAAGG
Fw-LMBD1-del848-851	GCATTTAGAATTCATTGAAAACAGCTGGTGG
Rv-LMBD1-del848-851	CTCTCTTCTTAAGTGTTCGTAACCTTTCTTC
Fw-mutLMBD1-GFP	ATCGATATGGTGAGCAAGGGCGAGGAGC
Rv-mutLMBD1-D469fs	AAAATACCCCAAGAAAGGCCCAGTTACCAAAATAG
Rv-mutLMBD1-K281fs	AATGCCTCTCTTCTTAAGTGTTCGTAACCTTTCTTC
Fw-LMBD1-233AAAA	CAGCTGCGGAAAACACTGAAGACATTGAAG

Rv-LMBD1-233AAAA	CAGCAGCAGCGCTTCTAGTGCCTTTTATC
Fw-LMBD1-294AAAA	CTTGTGGCGCTCTGCGTCCCCTGAAGATC
Rv-LMBD1-294AAAA	CTGCTGCCGCCCAGCTGTTTTCAATGAATTCTAAATGC
Fw-GFP- Cla	ATCGATATGGTGAGCAAGGGCGAGG
Rv-GFP- Cla	ATCGATTTACTTGTACAGCTCGTCCATGCC

Supplementary Figure Legends

Supplementary Figure 1 - Stable expression of LMBD1-GFP in CHO cells. PNS was prepared from CHO cells with or without the expression of LMBD1-GFP. LMBD1-GFP was detected by immunoblot analysis using an anti-GFP antibody.

Supplementary Figure 2 - Interaction between ABCD4 and LMBD1. ABCD4-HA constructs were transfected to CHO cells with or without the expression of LMBD1-GFP. Cell homogenates from the cells were treated with 1% digitonin and subjected to immunoprecipitation using an anti-GFP antibody. Co-precipitate proteins were analyzed by SDS-PAGE followed by immunoblotting using an anti-HA antibody.

Supplementary Figure 3 - Interaction between ABCD4-HA and ABCD4-His. ABCD4-His constructs were transfected to HuH7 cells with or without the expression of ABCD4-HA. Cell homogenates from the cells were treated with 1% digitonin and subjected to immunoprecipitation using an anti-HA antibody. Co-precipitated proteins were analyzed by SDS-PAGE followed by immunoblotting using an anti-His antibody.

Supplementary Figure 4 - Amino acid sequence alignment of ABCD4, ABCD1 and CmABCB1. Each of the shaded sequences was exchanged between ABCD4 and ABCD1.

Supplementary Figure 5 - Subcellular localization of LMBD1(K281fs)-GFP in HEK293 cells. The distribution of LMBD1(K281fs)-GFP was compared with that of ER or lysosomes labeled with anti-KDEL and anti-LAMP1, respectively. Bar, 20 μm.

Supplementary Figure 6 - Subcellular localization of ABCD4-HA in HEK293 cells

expressing mutant LMBD1-GFP. The distribution of ABCD4-HA and LMBD1(295WTKF/AAAA)-GFP was compared with that of lysosomes stained with anti-LAMP1. Bar, 20 μm.

Supplementary Figure 7 - Construction of *LMBRD1* knockout cells. (A) Sequence analysis of HEK293 cells injected with PX459-lmbrd1 targeting LMBRD1 gene. The PCR products were analyzed by direct sequencing to identify mutations in LMBRD1. (B) PNS was prepared from wild type and LMBRD1 knockout cells. LMBD1 was detected by immunoblot analysis using an anti-LMBD1 antibody (left) and an anti-Actin antibody (right). (C) Detection of endogenous ABCD4 in HEK293 cells. PNS (1.5 mg) from the wild type or LMBRD1 knockout cells was concentrated by Na_2CO_3 treatment. PNS (10 μg) of CHO cells expressing ABCD4 was used as the positive control. Endogenous ABCD4 was detected by immunoblot analysis using an anti-ABCD4 antibody. The asterisk indicates a non-specific signal.

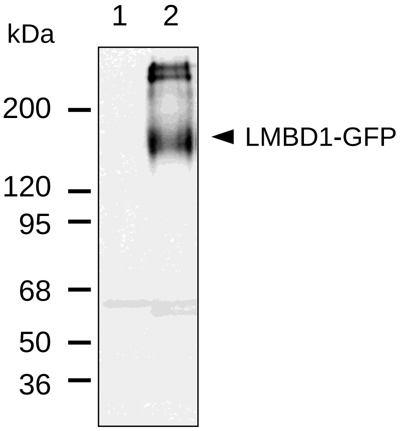

Kawaguchi et al. Figure S1

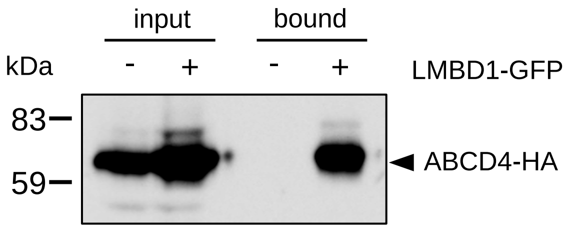

Kawaguchi et al. Figure S2

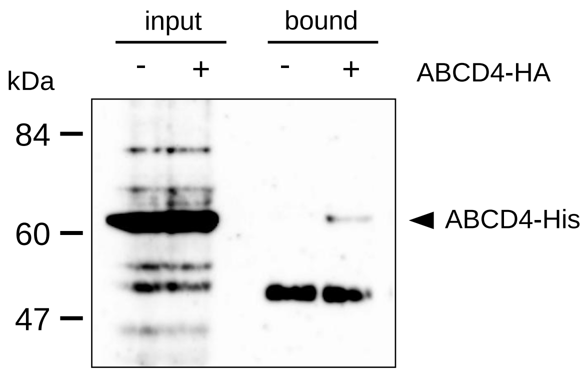

Kawaguchi et al. Figure S3

```
                                 TM1                                 TM2
CmABCB1   MNAHAAQSFETANYSSVGENHRERTLSSSIYETDPVSPDTAVSSEATAFQESFPSLKLKEKDTPNRRWWRFWARPSAAGEDPEAGDPKKAAKASGPESAY  100
HsABCD1   MPVLSRPRPWRGNTLKRTAVLL------ALAA----------YGAHKVYPLVRQCLAPARGLQAPAGEPTQEASGV-------AAAKAGMNRVF----LQ   73
HsABCD4   MAVAGPAPGAGARP--------------------------------------------------------------------------RLDLQF----LQ   22

CmABCB1   TTGVTARRIFALAWSSSATMIVIGFIASILEGATLPAFAIV-FGRMFQVFTKSKSQIEGETWKYSVGFVGIGVFEFIVAGSRTALFGIASERLARDLRVA  199
HsABCD1   RLLWLLRLLFPRVLCRETGLLALHSAALVSRTFLSVYVARLDGRLARCIVRKDPRAFGWQLLQWLLIALPATFVNSAIRYLEGQLALSFRSRLVAHAYRL  173
HsABCD4   RFLQILKVLFPSWSSQNALMFLTLLCLTLLEQFVIYQVGLIPSQYYGVLGNKDLEGFKTLTFLAVMLIVLNSTLKSFDQFTCNLLYVSWRKDLTEHLHRL  122
             chimera 1                                          chimera 2

                                 TM3                                 TM4
CmABCB1   AFSNLVEQDVTYFDRRK-------AGELGGKLNNDVQVIQYSFSKLGAVLFNLAQCVVGIIVAFIFAPALTGVLIALSPLVVLAGAAQMIEMSGNTKRSS  292
HsABCD1   YFSQQTYYRVSNMDGRLRNPDQSLTEDVVAFAASVAHLYSNLTKPLLDVAVTSYTLLRAARSRGAGTAWPSAIAGLVVFLTANVLRAFSPKFGELVAEEA  273
HsABCD4   YFRGRAYYTLNVLRDDIDNPDQRISQDVERFCRQLSSMASKLIISPFTLVYYTYQCFQSTGWLGP------VSIFGYFILGTVVNKTLMGPIVMKLVHQE  216
                          chimera 3                                                   chimera 4

                                 TM5
CmABCB1   EAYASAGSVAAEVFSNIRTTKAFEAERYETQRYGSKLDPLYRLGRRRYISDGLFFGLS-----MLVIFCVYALALWWGGQLIARGSLNL-----------  376
HsABCD1   RRKGELRYMHSRVVANSEEIAFYGGHEVELALLQRSYQDLASQINLILLERLWYVMLEQFLMKYVWSASGLLMVAVPIITATGYSESDAEAVKKAALEKK  373
HsABCD4   KLEGDFRFKHMQIRVNAEPAAFYRAGHVEHMRTDRRLQRLLQTQRELMSKELWLYIGI-NTFDYLGSILSYVVIAIPIFSGVY-----------------  298
                         chimera 5

                                 TM6
CmABCB1   --------------------------GNLLTAFFSAILGFMGVGQAAQVWPDVTRGLGAG----------GELFAMIDRVPQYRRPDPGAEVVTQPLVLK  440
HsABCD1   EEELVSERTEAFTIARNLLTAAADAIERIMSSYKEVTELAGYTARVHEMFQVFEDVQRCHFKRPRELEDAQAGSGTIGRSGVR--VEGPLKIRGQVVDVE  471
HsABCD4   GDLSPAELSTLVSKNAFVCIYLISCFTQLIDLSTTLSDVAGYTHRIGQLRETLLDMSLKSQDCE----------ILGE--SEW--GLDTPPGWPAAEPAD  384
             chimera 6

CmABCB1   QGIVFENVHFRYPTRMNVEVLRGISLTIPNGKTVAIVGGSGAGKSTIIQLLMRFYDIEPQGGGLLLFDGTPAWNYDFHALRSQIGLVSQEPVLFSGTIRD  540
HsABCD1   QGIICENIPIVTPSG--EVVVASLNIRVEEGMHLLITGPNGCGKSSLFRILGG---LWPTYGGVLYKPPPQ-----------RMFYIPQRPYMSVGSLRD  555
HsABCD4   TAFLLERVSISAPSS-DKPLIKDLSLKISEGQSLLITGNTGTGKTSLLRVLGG---LWTSTRGSVQMLTDF--------GPHGVLFLPQKPFFTDGTLRE  472
                                                   Walker A

CmABCB1   NILYGK-------RDATDEEVIQALREANAYSFVMALPDGLDTEVGERGLALSGGQKQRIAIARAILKHPTLLCLDESTSALDAESEALVQEALDRMMAS  633
HsABCD1   QVIYPDSVEDMQRKGYSEQDLEAILDVVHLHHILQREGGW--EAMCDWKDVLSGGEKQRIGMARMFYHRPKYALLDECTSAVSIDVEGK----IFQAAKD  649
HsABCD4   QVIYPLKEVYPDSGSADDERILRFLELAGLSNLVARTEGLDQQVDWNWYDVLSPGEMQRLSFARLFYLQPKYAVLDEATSALTEEVESE----LYRIGQQ  568
                                                                                      Walker B

CmABCB1   DGVTSVVIAHRLSTVARADLILVMQDGVVVEQGNHSELMALGPSGFYYQLVEKQLASGDMSAA-------------------------------------  696
HsABCD1   AGIALLSITHRPSLWKYHTHLLQFDGEG----GWKFEKLDSAA--RLSLTEEKQRLEQQLAGIPKMQRRLQELCQILGEAVAPAHVPAPSPQGPGGLQGA  743
HsABCD4   LGMTFISVGHRQSLEKFHSLVLKLCGGG----RWELMRIKVE----------------------------------------------------------  606
```

Kawaguchi et al. Figure S4

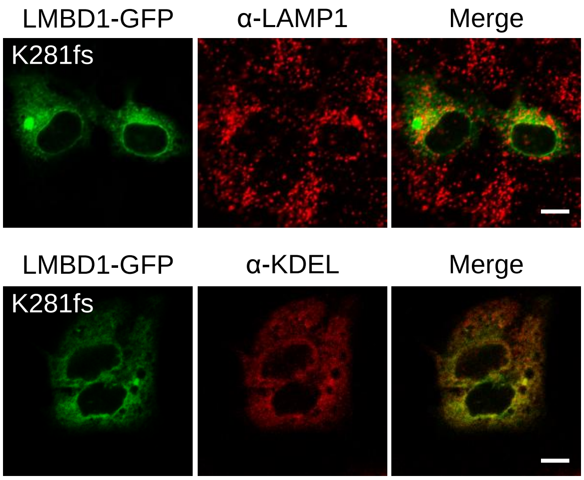

Kawaguchi et al. Figure S5

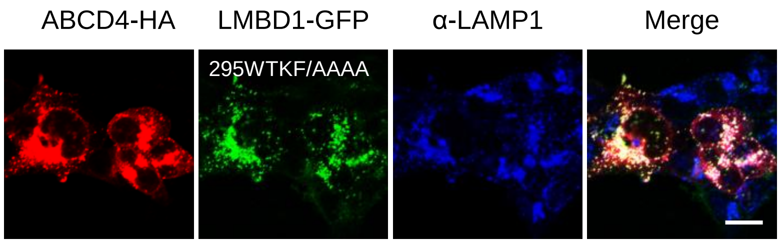

Kawaguchi et al. Figure S6

A

```
wild        TGGATATATGTTCGTAAATACCAAAGTCGGCGGGAAAGTGAAGTTGTCTC
knockout    TGGATATATGTTCGTAAAT-CC-------GCGGGAAAGATAAGGTGTCTC
```

B

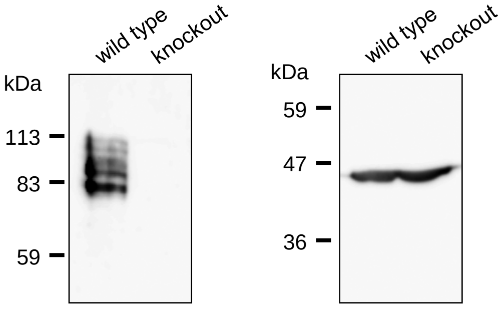

C

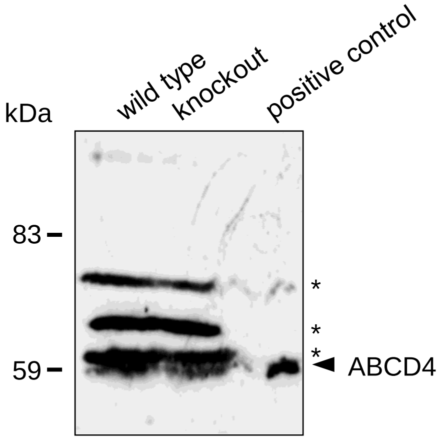

Kawaguchi et al. Figure S7
